# Mental health and psychosocial sequelae of fraud victimization: a systematic review

**DOI:** 10.3389/fpsyg.2026.1805536

**Published:** 2026-04-16

**Authors:** Jie Bai, Mei-Hsin Ho, Hsiang-Te Sung, Chuan-Sheng Hung, Jui-Hsiu Tsai

**Affiliations:** 1 Gannan Health Vocational College, Ganzhou, Jiangxi, China; 2 Department of Medical Research, Dalin Tzu Chi Hospital, Buddhist Tzu Chi Medical Foundation, Chiayi, Taiwan; 3 Department of Medical Education, Dalin Tzu Chi Hospital, Buddhist Tzu Chi Medical Foundation, Chiayi, Taiwan; 4 Gullas College of Medicine, Inc., University of the Visayas, Cibu, Philippines; 5 Department of Computer Science and Engineering, National Sun Yat-sen University, Kaohsiung, Taiwan; 6 Department of Psychiatry, Dalin Tzu Chi Hospital, Buddhist Tzu Chi Medical Foundation, Chiayi, Taiwan; 7 School of Medicine, Tzu Chi University, Hualien, Taiwan

**Keywords:** fraud, mental health, psychosocial outcomes, psychosocial sequelae, victimization

## Abstract

Fraud victimization is an increasingly prevalent global problem that extends beyond financial loss, and encompasses substantial mental health and psychosocial harm. Deception, emotional manipulation, and perceived betrayal have been linked to anxiety, depression, shame, trust erosion, and reduced quality of life. Despite these effects, the psychological consequences of fraud victimization remain under-recognized within healthcare and victim support systems. This systematic review synthesizes empirical evidence of mental health and psychosocial outcomes among adults following fraud or scam victimization. A systematic literature search was conducted in accordance with Preferred Reporting Items for Systematic Reviews and Meta-Analyses (PRISMA) guidelines across four electronic databases (PubMed, OVID, Web of Science, and Embase) from inception to December 31, 2025. Empirical quantitative, qualitative, and mixed-methods studies examining post-victimization mental health or psychosocial outcomes among adult fraud victims were included. Studies exclusively exploring fraud risk, susceptibility, or financial and legal outcomes without assessing the psychological effects of post-victimization were excluded. Owing to heterogeneity in study designs and outcome measures, the findings were synthesized using a narrative approach. Twenty-one studies met the inclusion criteria. Across diverse populations, fraud types, and cultural contexts, fraud victimization was associated with adverse mental health outcomes, including elevated anxiety, depression, psychological distress, shame, self-blame, and diminished quality of life. Psychological harm frequently persisted beyond the resolution of financial losses and was more strongly associated with emotional manipulation, perceived betrayal, and interpersonal trust erosion than with the magnitude of financial loss alone. This systematic review demonstrates that fraud victimization is associated with substantial and enduring mental health and psychosocial sequelae. The findings support reframing fraud victimization as a public mental health concern and underscore the need to integrate trauma-informed and psychosocial care into fraud responses and victim support services. Future research should prioritize longitudinal and intervention studies to elucidate recovery trajectories and inform effective mental health responses for fraud victims.

## 1 Introduction

Fraud and scam victimization have emerged as significant and growing societal challenges, affecting individuals across age groups and regions, resulting in substantial financial and psychosocial harm. Population-level evidence indicates that fraud is highly prevalent, particularly among older adults, and increasingly represents a public health concern beyond criminal or economic domains (Burnes et al., 2017; Button et al., 2025). Fraud refers to intentional deceptive practices in which individuals are misled for financial or personal gain (Levi and Burrows, 2008; Button et al., 2014). Within this broad framework, scams are generally conceptualized as a subset of fraud, often involving interpersonal or technologically mediated forms of deception. These fraudulent practices encompass several types of victimization, including financial fraud, identity theft, mass-marketing fraud, and various forms of online scams such as romance scams. Despite differing in their operational mechanisms and interpersonal dynamics, these modalities share a common underlying element of deception, which may result in adverse psychological and psychosocial consequences following victimization. Conceptual and empirical research further demonstrates that the consequences of fraud extend beyond direct financial loss to broader disruptions in well-being and social functioning (Levi and Burrows, 2008; Kassem, 2024).

Fraud victimization is inherently emotionally salient, as they frequently involve deception, manipulation, and violations of interpersonal trust. Research on fraud and cybercrime victimization shows that fraud victimization can elicit intense emotional responses, including fear, anxiety, psychological distress, shame, and self-blame, which may persist well beyond the fraudulent events (Buchanan and Whitty, 2014; Borwell et al., 2025). Importantly, these emotional responses are shaped by material loss and the relational and psychological dynamics embedded within fraudulent interactions.

Psychologically, fraud victimization parallels betrayal-based and interpersonal trauma, where violations of trust and autonomy may produce lasting emotional and cognitive consequences (Freyd, 1998). Self-conscious emotions, such as shame and guilt, are particularly salient following fraud victimization, as individuals may internalize blame and perceive the experience as a personal failure (Tangney and Dearing, 2003). These responses are often exacerbated by social stigma and anticipated judgment, contributing to concealment, delayed disclosure, and barriers to seeking help (Corrigan, 2004; Clement et al., 2015).

Although research has increasingly examined the psychological dimensions of fraud, most existing studies explore risk factors, susceptibility, emotional processes during scam engagement, and compliance mechanisms, rather than post-victimization mental health outcomes (Kennedy et al., 2021; Norris and Brookes, 2021; Wen et al., 2022). Existing reviews highlight that, although psychological harm is frequently acknowledged, empirical evidence of post-victimization mental health and psychosocial sequelae is fragmented across disciplines and heterogeneous in study design, populations, and outcome measures (Norris et al., 2019; Shang et al., 2022).

Recent academic syntheses and commentaries have identified critical gaps in understanding how fraud victimization affects individuals’ well-being over time, particularly regarding psychological distress, emotional adjustment, and social functioning (Kassem, 2024; Balcombe, 2025). Simultaneously, clinical and practice-oriented literature emphasizes the need for greater recognition of the psychosocial needs of fraud victims, especially among older adults, while underscoring the limited integration of mental health perspectives into existing fraud response systems (Lichtenberg et al., 2019).

Notably, fraud victimization encompasses heterogeneous forms of deception, which vary in relational dynamics, emotional manipulation, and financial mechanisms. For example, romance scams often involve prolonged interpersonal interaction and emotional manipulation, whereas identity theft or financial fraud may occur through more transactional mechanisms (Whitty and Buchanan, 2016; Cole, 2024).

Accordingly, there is a clear need for a comprehensive synthesis of empirical evidence examining the mental health and psychosocial sequelae of fraud and scam victimization. This systematic review synthesized empirical quantitative and qualitative studies assessing post-victimization among adults, focusing on common psychosocial impacts across fraud modalities. By consolidating this evidence, the review aimed to elucidate the scope of psychological harm and contribute to trauma-informed, stigma-sensitive clinical, social, and public health interventions.

## 2 Methods

### 2.1 Search strategy

This systematic review was conducted in accordance with the Preferred Reporting Items for Systematic Reviews and Meta-Analyses (PRISMA) guidelines. A comprehensive literature search was performed across four electronic databases (PubMed, OVID, Web of Science, and Embase) from database inception to December 31, 2025.

Search terms related to fraud victimization (e.g., *“fraud*,” “scam*,” “fraud victimization,” “financial fraud,” “online fraud,” “mass marketing fraud”*) were combined with terms related to mental health and psychosocial outcomes (e.g., *“mental health,” “psychological distress,” “depression,” “anxiety,” “stress,” “trauma,” “well-being,”* “quality of life”). Searches were applied to titles, abstracts, and keywords.

Additionally, the reference lists of all included articles were manually screened to identify relevant studies not captured through the database searches. The primary outcomes of interest encompassed post-victimization mental health and psychosocial outcomes, including psychological distress, emotional and affective responses, social and interpersonal consequences, and quality of life.

### 2.2 Inclusion criteria

Inclusion criteria were defined *a priori* to ensure alignment with the study objectives. The inclusion criteria were:

1. Publication period: Studies published up to December 2025, with no lower limit on the publication year.
2. Population: Adults aged 18 years or older who had experienced fraud or scam victimization. Studies that included broader populations were considered eligible if outcomes specific to fraud victims could be identified or extracted.
3. Exposure: Fraud or scam victimization, including but not limited to financial fraud, online fraud, romance scams, and mass-marketing fraud.
4. Study design: Empirical quantitative, qualitative, and mixed-methods studies. Mixed-methods studies were included if mental health or psychosocial outcomes were explicitly reported.
5. Outcomes: Studies assessing at least one mental health or psychosocial outcome after fraud victimization, such as anxiety, depression, psychological distress, trauma-related symptoms, shame, self-blame, changes in interpersonal trust, coping responses, or quality of life. Outcomes were required to be assessed using validated instruments, structured interviews, or clearly defined qualitative methodologies.
6. Language: Peer-reviewed articles published in English.

### 2.3 Exclusion criteria

The exclusion criteria were:

1. Studies were excluded if fraud or scam victimization was not the primary outcome or if mental health outcomes were not assessed.
2. Exclusively explored the technical aspects of fraud detection, cybersecurity systems, algorithm development, and financial mechanisms, excluding victims’ mental health or psychosocial outcomes.
3. Addressed only legal, regulatory, or financial processes related to fraud, excluding psychological or psychosocial consequences.
4. Review articles, meta-analyses, editorials, commentaries, letters, opinion papers, or conference abstracts lacking full-text empirical data.
5. Consisted solely of single case reports or small case series that did not allow for generalizable conclusions.
6. Lacked sufficient data for extraction and meaningful analysis (e.g., unclear outcome measures or incomplete reporting).
7. Primarily explored vulnerability, susceptibility, personality traits, or risk factors for fraud victimization, excluding mental health or psychosocial outcomes of victimization.

### 2.4 Search process results

**Figure 1:**
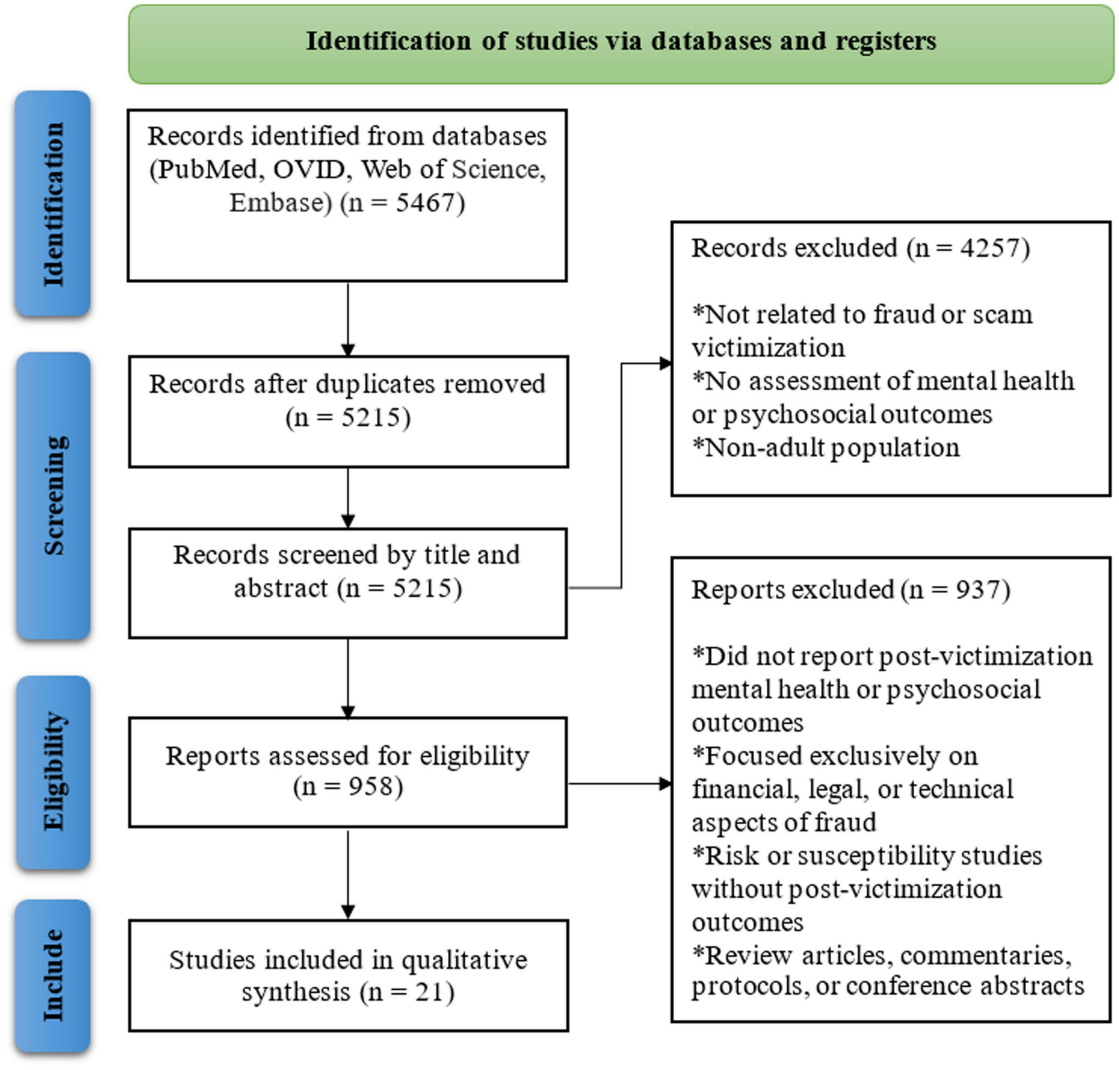
Flowchart showing the literature search and article selection process used by the authors to identify studies on mental health and psychosocial sequelae following fraud victimization.

The literature search identified 5,467 records across four electronic databases (PubMed, OVID, Web of Science, and Embase). After eliminating duplicate records, 5,215 records remained and were screened by title and abstract; of these, 4,257 were excluded. Full-text reports of 958 articles were assessed for eligibility, and 937 articles were excluded for not reporting post-victimization mental health or psychosocial outcomes, exclusively exploring financial, legal, or technical aspects of fraud, addressing fraud risk or susceptibility without post-victimization outcomes, or meeting other exclusion criteria. Twenty-one studies were included in the final qualitative synthesis. The PRISMA flow diagram (Figure 1) summarizes the study selection process.

## 3 Results

Twenty-one empirical studies examining mental health or psychosocial outcomes following fraud or scam victimization were included in the final synthesis. As summarized in Table 1, the included studies employed quantitative, qualitative, and mixed-methods designs and represented diverse populations, fraud modalities, and geographic regions. All included studies explicitly assessed mental health or psychosocial sequelae of fraud victimization. Studies solely exploring risk factors, susceptibility, or predictors of fraud exposure were excluded in accordance with the predefined inclusion criteria.

**Table 1:** Characteristics of studies examining post-victimization mental health and psychosocial sequelae following fraud or scam victimization.

Author (Year)	Country/Region	Study design	Population	Type of fraud	Post-victimization mental health/Psychosocial outcomes	Key findings
Sarriá et al. (2019)	Spain (Madrid)	Cross-sectional survey (*n* = 4,425)	General population	Financial fraud	Depression, anxiety, QoL (COOP/WONCA)	Fraud victims reported poorer mental health and reduced quality of life, independent of financial loss (F > M)
DeLiema et al. (2021)	USA	Survey analysis (*n* = 2,513)	Older adults	Identity theft	Psychological distress, financial strain	Identity theft associated with sustained psychological distress and financial burden
Golladay and Holtfreter (2017)	USA	Quantitative survey (*n* = 8)	Adult victims	Identity theft	Emotional distress, physical health complaints	Victimization linked to emotional distress and adverse physical health outcomes
Kim et al. (2025)	South Korea	Population-based analysis (*n* = 22,483–36,008)	Older adults	Fraud crime	Suicide risk	Fraud exposure incremental associations with suicide risk among older adults
Wang et al. (2023)	China	Cross-sectional study (*n* = 15,322)	Middle-aged and older adults	Financial fraud	Depressive symptoms (CES-D10)	Defraudation was significantly associated with higher depressive symptoms
Weissberger (2022)	Israel	Cross-sectional survey (*n* = 137)	Older adults	Financial exploitation	Anxiety, depression (CES-D, GAD-7)	Higher anxiety and depressive symptoms among exploited individuals
Weissberger et al. (2020)	USA	Observational study (*n* = 103)	Older adults	Financial exploitation	Mental and physical health status	Perceived exploitation associated with poorer overall health
Button et al. (2021)	UK	Qualitative interviews (*n* = 52)	Adult victims	Computer misuse crime	Trauma, shame, distress	Emotional effects ranged from severe trauma to minimization
Wang et al. (2024)	China	Text-based qualitative analysis (*n* = 50)	Online fraud victims	Online fraud	Dynamic emotional trajectories	Emotional experiences evolved from trust to confusion, distress, and anger
Whitty and Buchanan (2016)	UK/Australia	Mixed-methods (*n* = 20)	Online dating users	Romance scam	Psychological distress, trust erosion	Non-financial effects often exceeded financial losses
Button et al. (2014)	UK	Qualitative interviews (≈800 calls)	Adult victims and families	Financial fraud	Emotional distress, family strain	Fraud negatively affected emotional well-being and family relationships
Cross et al. (2016)	Australia	Qualitative interviews (*n* = 80)	Online fraud victims	Online fraud	Distress, unmet support needs	Shame and barriers to disclosure delayed help-seeking
Cole (2024)	USA	Qualitative study (*n* = 19)	Romance scam victims	Online romance scams	Emotional, physiological stress	Victims experienced long-term emotional and legal consequences
Deem (2000)	USA	Field observations (*n* = 325)	Older adults	Personal financial crime	Psychological distress, emotional vulnerability	Older victims experienced substantial but often overlooked psychological harm
Ganzini et al. (1990a)	USA	Epidemiological study (*n* = 72)	Adults	Catastrophic financial loss	Mental disorders (MDD, GAD)	High prevalence of psychiatric disorders following severe financial loss
Ganzini et al. (1990b)	USA	Comparative study (*n* = 77)	Fraud vs. violent crime victims	Fraud	Psychological impact (MDD, GAD)	Psychological harm comparable to victims of violent crime
Gould et al. (2023)	Australia	Qualitative study (*n* = 7 survivors, 6 caregivers)	ABI survivors	Cyberscams	Shame, emotional distress	Cognitive impairment amplified stigma and emotional harm
Kemp and Erades Pérez (2023)	Europe Union	Survey study (*n* = 26,735)	Older adults	Consumer fraud	Mental well-being decline	Digital consumer fraud associated with poorer mental well-being
Xia et al. (2025)	China	Longitudinal survey (*n* = 21,803)	General population	Fraud victimization	Erosion of generalized social trust	Fraud experiences predicted reduced generalized trust over time
Zeghiche et al. (2023)	Canada	Qualitative study (*n* = 15)	Families	Insemination fraud	Family relationship strain	Fraud disrupted trust and family dynamics
Zunzunegui et al. (2017)	Spain	Population-based study (*n* = 188)	Older adults	Financial fraud	Self-rated health, sleep quality, quality of life	Fraud associated with poorer self-rated health and well-being

Most quantitative studies used population-based or survey designs to examine post-victimization psychological distress, depressive and anxiety symptoms, health status, and quality of life. Qualitative and mixed-methods studies provided in-depth accounts of emotional, relational, and psychosocial consequences, including shame, self-blame, interpersonal trust erosion, and disruptions in family and social functioning. Given the substantial heterogeneity in study designs, populations, fraud types, and outcome measures, findings were synthesized using a narrative approach. Table 2 presents a regional synthesis of predominant fraud types and psychological responses.

**Table 2:** Regional classification of fraud types and post-victimization psychological responses.

Region	Countries represented (Table 1)	Predominant fraud/Scam types	Core psychological and psychosocial responses	Representative studies
North America	USA, Canada	Identity theft; Financial exploitation of older adults; Online romance scams; Catastrophic financial loss	Depression and anxiety (clinical and subclinical); Psychological distress and trauma-like symptoms; Shame and self-blame; Physical health deterioration; Long-term emotional and legal stress; Family strain and social withdrawal	DeLiema et al. (2021), Golladay and Holtfreter (2017), Ganzini et al. (1990a, 1990b), Cole (2024), Zeghiche et al. (2023)
Europe	Spain, United Kingdom	Financial fraud; Computer misuse crime; Romance scams	Depressive and anxiety symptoms; Reduced quality of life and self-rated health; Shame, embarrassment, and emotional distress; Trauma responses ranging from severe distress to minimization; interpersonal trust erosion	Sarriá et al. (2019), Zunzunegui et al. (2017), Button et al. (2014, 2021), Whitty and Buchanan (2016), Kemp and Erades Pérez (2023)
Western Asia	Israel	Financial exploitation; Financial fraud	Anxiety and depressive symptoms; Psychological distress; Poorer perceived mental and physical health; Psychosocial vulnerability among older adults	Weissberger (2022), Weissberger et al. (2020)
Asia	China, South Korea	Financial fraud; Online fraud; General fraud crime	Depressive symptoms; Dynamic emotional trajectories (trust → confusion → distress/anger); Reduced generalized social trust; Increased suicide risk reported in population-based studies	Wang et al. (2023), Wang et al. (2024), Kim et al. (2025), Xia et al. (2025)
Oceania	Australia	Online fraud; Cyberscams	Psychological distress and shame; Stigma-related self-blame; Barriers to disclosure and help-seeking; Social and emotional burden among vulnerable groups	Cross et al. (2016), Gould et al. (2023), Whitty and Buchanan (2016)

### 3.1 Mental health outcomes of fraud victimization

Across the included studies, fraud victimization was associated with adverse mental health outcomes. As summarized in Table 2, heightened psychological distress, depressive symptoms, and anxiety were reported across North America, Europe, Western Asia, and Asia, despite heterogeneous fraud modalities and study populations. Multiple studies documented elevated psychological distress, depressive symptoms, and anxiety among fraud victims across different fraud types and populations (Zunzunegui et al., 2017; Golladay and Holtfreter, 2017; Sarriá et al., 2019; Weissberger et al., 2020; DeLiema et al., 2021; Weissberger, 2022; Kemp and Erades Pérez, 2023; Wang et al., 2023).

Population-based studies further documented these associations. In Spain, financial fraud victims reported significantly poorer self-rated and mental health than their counterparts, independent of the magnitude of financial loss (Zunzunegui et al., 2017; Sarriá et al., 2019). In China, defrauded middle-aged and older adults reported elevated depressive symptoms (Wang et al., 2023). Similarly, studies from Israel and the United States linked perceived financial exploitation to elevated anxiety, depressive symptoms, and poor overall mental health (Weissberger et al., 2020; DeLiema et al., 2021; Weissberger, 2022). Research on older adults additionally reported emotional distress, diminished trust, and increased psychosocial needs following financial exploitation (Weissberger et al., 2020; DeLiema et al., 2021; Kemp and Erades Pérez, 2023).

Severe or catastrophic financial loss was associated with clinically significant psychiatric outcomes, particularly in population-based studies. Population-based evidence demonstrated a higher prevalence of depression, anxiety, and substance-related disorders among individuals experiencing catastrophic financial loss (Ganzini et al., 1990a). As summarized in Table 1, these findings represent the severe end of post-victimization mental health outcomes. Comparative analyses further indicated that fraud victims exhibited psychiatric distress comparable to, and in some domains exceeding, that reported by victims of violent crime (Ganzini et al., 1990b).

Qualitative and mixed-methods studies documented trauma-like responses, including persistent worry, intrusive thoughts, heightened arousal, and difficulty regaining a sense of safety, particularly following prolonged or emotionally manipulative fraud such as online romance scams (Whitty and Buchanan, 2016; Cole, 2024; Wang et al., 2024).

### 3.2 Emotional processes and psychosocial consequences

Fraud victimization elicited complex emotional responses and psychosocial consequences. Victims often experienced shame, embarrassment, and self-blame, internalizing responsibility and interpreting victimization as a personal failure (Button et al., 2014; Whitty and Buchanan, 2016; Deem, 2000; Gould et al., 2023). Such internalized blame was linked to diminished self-worth, reduced confidence, and reluctance to disclose victimization, with fear of judgment and stigma frequently delaying help-seeking (Cross et al., 2016; DeLiema et al., 2021; Gould et al., 2023).

Emotional experiences evolved dynamically over the course of fraudulent interactions. Initial trust, hope, or excitement often shifted to confusion, anxiety, and self-reproach, a process exploited by perpetrators to reinforce compliance (Whitty and Buchanan, 2016; Cole, 2024; Wang et al., 2024). Negative emotional responses also contributed to prolonged engagement with perpetrators, amplifying psychological strain and distress (Cross et al., 2016).

Socially, fraud victimization eroded interpersonal trust and strained relationships. Victims reported heightened suspicion, difficulties maintaining relationships, and tension or conflict within families and social networks (Button et al., 2014; Whitty and Buchanan, 2016; Zeghiche et al., 2023; Xia et al., 2025).

### 3.3 Impact on well-being, social functioning, and cumulative burden

Included studies reported a decline in well-being and quality of life following fraud victimization. Population-based surveys and large-scale observational studies, summarized in Table 1, documented lower self-rated health and life satisfaction among fraud victims, with similar patterns observed across multiple regions (Table 2). In Spain, population-based studies reported poorer self-rated health, mental health, and life satisfaction among financial fraud victims, even when financial losses were modest (Zunzunegui et al., 2017; Sarriá et al., 2019). Diminished mental well-being and quality of life were also observed among older adults exposed to digital consumer fraud (Kemp and Erades Pérez, 2023).

Qualitative studies described disruptions in daily functioning following fraud victimization, including social withdrawal, avoidance of financial or digital environments, and reduced confidence in personal safety, often in the context of shame, self-blame, and trust erosion (Cross et al., 2016; Deem, 2000; Cole, 2024).

Several studies reported more pronounced effects among individuals experiencing repeated or prolonged fraud victimization. These individuals described more persistent emotional distress, greater social dysfunction, and sustained reductions in well-being (DeLiema et al., 2021). In population-based studies from Asia, fraud victimization was associated with increased suicide risk among older adults (Kim et al., 2025).

## 4 Discussion

### 4.1 Principal findings and conceptual implications

Unlike prior reviews that primarily focused on specific fraud types (e.g., romance scams), this review integrated evidence across multiple fraud modalities to identify shared psychosocial mechanisms and outcomes. By adopting a cross-modality perspective, this review extends the literature by providing a broader conceptualization of fraud victimization as a mental health concern. Drawing on the empirical evidence summarized in Table 1 and the cross-regional synthesis presented in Table 2, this systematic review demonstrated that fraud victimization is associated with substantial and multifaceted mental health and psychosocial consequences. Across quantitative, qualitative, and mixed-methods studies, victims consistently reported elevated psychological distress, depressive and anxiety symptoms, shame, interpersonal trust erosion, and reduced quality of life (Buchanan and Whitty, 2014; Button et al., 2014; Borwell et al., 2025). These outcomes were observed across diverse fraud modalities, age groups, and methodological approaches, indicating that post-victimization psychological harm is neither incidental nor confined to specific populations.

The studies included in this review examined diverse fraud modalities, including financial fraud, identity theft, online scams, and romance scams. These fraud types differ in their operational mechanisms and interpersonal dynamics (Levi and Burrows, 2008). For example, romance scams often involve prolonged emotional manipulation and perceived betrayal through sustained interpersonal interaction, whereas transactional fraud such as identity theft or financial fraud may occur with limited or no direct interpersonal contact (Whitty and Buchanan, 2016; Cole, 2024). Given this heterogeneity, this review did not assume equivalence across fraud modalities; the findings should be interpreted as cross-cutting psychosocial patterns rather than uniform effects. Despite these differences, the evidence synthesized in this review suggests that multiple fraud modalities share common psychological consequences following victimization, including distress, shame, and diminished trust (Button et al., 2014).

Population-based studies summarized in Table 1 provide consistent evidence that fraud victimization is associated with poorer self-rated mental health, increased depressive symptoms, and diminished well-being, even after accounting for sociodemographic factors and, in several studies, independent of the magnitude of financial loss (Zunzunegui et al., 2017; Sarriá et al., 2019; Wang et al., 2023). Complementing these findings, qualitative and mixed-methods studies documented emotionally salient and relational consequences, including shame, self-blame, trauma-like responses, and disruptions in family and social functioning, which often persisted beyond the resolution of financial losses (Button et al., 2014; Whitty and Buchanan, 2016; Cole, 2024).

The regional synthesis in Table 2 indicates substantial convergence in psychological responses across North America, Europe, Western Asia, Asia, and Oceania. Despite variation in fraud types, cultural contexts, and measurement strategies, similar patterns of emotional distress, trust erosion, and reduced well-being were observed. Collectively, these findings support the conceptualization of fraud victimization as a financial or legal transgression and a psychologically meaningful form of victimization with enduring implications for mental health and psychosocial functioning (Levi and Burrows, 2008; Kassem, 2024; Balcombe, 2025).

### 4.2 Psychological mechanisms underlying post-victimization distress

The psychological sequelae identified across the studies in Table 1 can be interpreted through self-regulation and affective decision-making models, emphasizing the roles of emotional arousal, cognitive load, and diminished regulatory capacity during deceptive interactions (Bauer and Baumeister, 2011; Wen et al., 2022). Fraud schemes commonly involve urgency, emotional engagement, and interpersonal manipulation, imposing substantial demands on cognitive control and intensifying emotional investment. When victimization is later recognized, these emotional processes may contribute to intensified distress, regret, and self-reproach.

Dual-process theories further suggest that emotionally charged contexts bias individuals toward intuitive and affect-driven processing, potentially constraining deliberative evaluation and complicating post-event cognitive reappraisal (Kahneman, 2011). The empirical studies summarized in Table 1 consistently reported persistent distress, intrusive rumination, and difficulty integrating the experience into coherent self-narratives following fraud victimization (Norris and Brookes, 2021; Wang et al., 2024; Gauci and Vella, 2025). Qualitative analyses further described dynamic emotional trajectories in which initial trust, hope, or excitement shifted toward confusion, shame, and emotional exhaustion (Whitty and Buchanan, 2016; Wang et al., 2024). Additionally, large-scale analyses of victim self-reports indicated that emotional reactions such as distress, anger, regret, and shame are salient features of post-victimization, reinforcing the role of emotional processes as a pathway linking deceptive interactions to long-term psychological harm (DeLiema and Witt, 2024).

Notably, these emotional processes were not merely antecedents to fraud exposure but were repeatedly reported as emerging from the victimization process. Studies exploring emotionally manipulative fraud, particularly online romance scams, emphasized that prolonged interpersonal engagement and perceived betrayal were central to long-term psychological harm, even after financial losses had been addressed (Balcombe, 2025; Borwell et al., 2025). In rare but severe cases, extreme psychological sequelae, including psychotic symptoms, have been reported in contexts of intense emotional manipulation (Alotti et al., 2024), highlighting the potential severity of post-victimization distress.

### 4.3 Shame, stigma, and social processes following victimization

Shame and self-blame emerged as central psychosocial processes shaping post-victimization experiences across regions and fraud types, as evidenced by qualitative and population-based studies from Europe, North America, Asia, and Oceania (Table 2). Victims often internalized blame, perceiving victimization as a personal failure rather than deliberate exploitation, which was associated with diminished self-worth and confidence (Buchanan and Whitty, 2014; Tangney and Dearing, 2003).

Social stigma further compounded these emotional responses. Consistent with stigma frameworks, fear of judgment and anticipated social disapproval were commonly reported as barriers to disclosure and seeking help, thereby limiting access to social validation and formal support (Corrigan, 2004; Clement et al., 2015). The qualitative studies summarized in Table 1 indicated that delayed reporting, concealment, and social withdrawal were frequent, particularly among emotionally manipulative scam victims, such as those experiencing romance fraud (Whitty and Buchanan, 2016; Cross and Lee, 2022; Gould et al., 2023; DeLiema and Witt, 2024).

These social and emotional processes may sustain psychological distress beyond the initial fraud. Across regions (Table 2), shame and self-blame were consistently associated with prolonged distress, strained family relationships, and disruptions in social functioning, suggesting that stigma-related mechanisms influence recovery trajectories following fraud victimization (Button et al., 2014; Borwell et al., 2025).

### 4.4 Beyond financial loss: betrayal, trust, and well-being

The findings summarized in Table 1 challenge the purely economic interpretations of fraud victimization. Across multiple population-based studies, the magnitude of financial loss was weakly or inconsistently associated with psychological harm, whereas perceived betrayal, emotional manipulation, and trust erosion emerged as more consistent correlates of post-victimization distress (Zunzunegui et al., 2017; Sarriá et al., 2019). These findings align with betrayal trauma frameworks, which emphasize violations of trust, autonomy, and relational expectations as central determinants of psychological injury (Freyd, 1998).

Fraud frequently exploits interpersonal trust through impersonation, sustained communication, or romantic deception, thereby undermining victims’ assumptions about others’ reliability and the safety of social and digital environments. Evidence synthesized across regions in Table 2 indicates that fraud victimization is associated with diminished generalized trust, heightened suspicion, and perceived loss of social safety, contributing to broader disruptions in social functioning and perceived control (Thoits, 2011; Weissberger and Bergman, 2025; Xia et al., 2025).

From this perspective, diminished well-being following fraud victimization reflects emotional distress as well as lasting changes in interpersonal orientation and quality of life (Diener et al., 1999; Kassem, 2024). By integrating study-level and regional evidence, this review indicates that the psychological impact of fraud is shaped less by financial damage than by the relational and emotional dynamics of deception.

### 4.5 Clinical and public health implications

Despite growing evidence of psychological harm, current responses to fraud victimization remain largely oriented toward financial restitution and legal remedies, with limited integration of mental health or psychosocial care. Qualitative studies also suggest that victims’ experiences when reporting fraud to institutions such as banks or law enforcement agencies may influence psychological recovery. Dismissive responses, perceived blame, or bureaucratic barriers may exacerbate distress and discourage help-seeking among victims (Cross et al., 2016; Button et al., 2014). The findings of this review highlight the need for trauma-informed and stigma-sensitive approaches that explicitly address emotional distress, shame, and barriers to seeking help (Corrigan, 2004; Clement et al., 2015; Balcombe, 2025). Incorporating routine mental health screening, psychoeducation, and referral pathways into fraud response services may facilitate earlier identification of individuals at risk of prolonged psychological distress.

At the population level, the evidence synthesized in this review supports reframing fraud victimization as a public mental health concern. The accumulated evidence may inform the development of integrated policies that extend beyond prevention and enforcement to include psychosocial recovery and support. Such an approach aligns with recent calls to recognize the broader societal and health outcomes of fraud, particularly in aging and digitally mediated societies (Kassem, 2024; Button et al., 2025).

The process of seeking financial restitution or legal resolution may constitute an additional stressor, particularly when victims encounter complex procedures, delayed responses, or perceived invalidation. However, this aspect remains underexplored in the literature and warrants further investigation.

### 4.6 Strengths, limitations, and future directions

This review synthesizes evidence of the mental health and psychosocial consequences of fraud victimization across diverse populations, fraud types, and cultural contexts. Limitations include the predominance of cross-sectional and qualitative designs, which constrain causal inference and the understanding of recovery trajectories. Additionally, rare and severe psychiatric outcomes were primarily documented in case-based studies, limiting generalizability. The limited reporting of suicide-related outcomes in the reviewed literature may reflect methodological constraints, as most studies adopted cross-sectional survey designs assessing general psychological distress rather than rare psychiatric outcomes. Detecting suicide risk may require large population-based datasets or longitudinal follow-up. Heterogeneity in outcome measures further complicates comparisons across studies.

Future research should prioritize longitudinal and intervention studies to elucidate the temporal course of psychological recovery and evaluate the effectiveness of trauma-informed psychosocial interventions. In particular, studies examining emotional processes, betrayal dynamics, and stigma may help clarify recovery trajectories and inform strategies for mitigating post-victimization psychological harm. Moreover, research in underrepresented and non-Western contexts is needed to strengthen the global relevance of this evidence base and support the development of culturally sensitive support systems for fraud victims.

## 5 Conclusion

Fraud victimization is associated with substantial mental health and psychosocial harm, which extends beyond financial loss, encompassing emotional manipulation, perceived betrayal, and lasting trust erosion; emotional regulation; and overall well-being. Psychological consequences are often not proportional to monetary damage, with shame, self-blame, and trust erosion shaping disclosure, help-seeking, and recovery trajectories. Despite the growing recognition of these harms, institutional responses to fraud victimization continue to prioritize financial restitution and legal remedies, while mental health and psychosocial support remain comparatively underdeveloped. These findings underscore the importance of trauma-informed and stigma-sensitive approaches to address the emotional and relational dimensions of deception.

## Data Availability

The original contributions presented in the study are included in the article/supplementary material, further inquiries can be directed to the corresponding author.
