# Correction: Mental health and psychosocial sequelae of fraud victimization: a systematic review

**DOI:** 10.3389/fpsyg.2026.1858733

**Published:** 2026-04-29

**Authors:** Jie Bai, Mei-Hsin Ho, Hsiang-Te Sung, Chuan-Sheng Hung, Jui-Hsiu Tsai

**Affiliations:** 1 Gannan Health Vocational College, Ganzhou, Jiangxi, China; 2 Department of Medical Research, Dalin Tzu Chi Hospital, Buddhist Tzu Chi Medical Foundation, Chiayi, Taiwan; 3 Department of Medical Education, Dalin Tzu Chi Hospital, Buddhist Tzu Chi Medical Foundation, Chiayi, Taiwan; 4 Gullas College of Medicine, Inc., University of the Visayas, Cibu, Philippines; 5 Department of Computer Science and Engineering, National Sun Yat-sen University, Kaohsiung, Taiwan; 6 Department of Psychiatry, Dalin Tzu Chi Hospital, Buddhist Tzu Chi Medical Foundation, Chiayi, Taiwan; 7 School of Medicine, Tzu Chi University, Hualien, Taiwan

**Keywords:** fraud, mental health, psychosocial outcomes, psychosocial sequelae, victimization

An outdated version of Figure 1 was erroneously published in the article. The corrected Figure 1 appears below.

**Figure 1:**
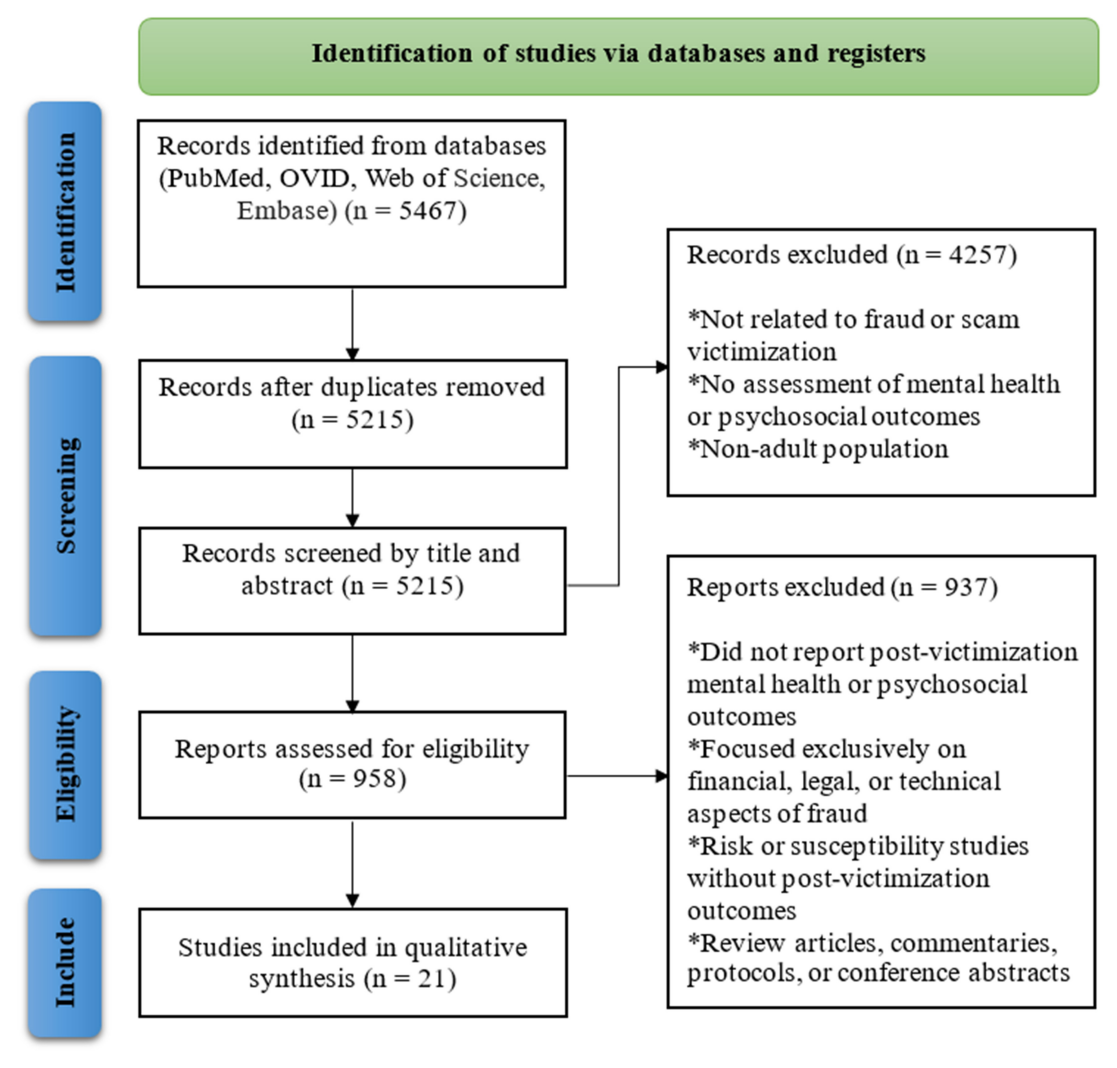
Flowchart showing the literature search and article selection process used by the authors to identify studies on mental health and psychosocial sequelae following fraud victimization.

The original version of this article has been updated.

